# 2-D Minimum Variance Based Plane Wave Compounding with Generalized Coherence Factor in Ultrafast Ultrasound Imaging

**DOI:** 10.3390/s18124099

**Published:** 2018-11-23

**Authors:** Yanxing Qi, Yuanyuan Wang, Jinhua Yu, Yi Guo

**Affiliations:** 1 Department of Electronic Engineering, Fudan University, Shanghai 200433, China; 17110720012@fudan.edu.cn (Y.Q.); jhyu@fudan.edu.cn (J.Y.); guoyi@fudan.edu.cn (Y.G.); 2 Key Laboratory of Medical Imaging Computing and Computer Assisted Intervention (MICCAI) of Shanghai, Shanghai 200032, China

**Keywords:** ultrafast ultrasound imaging, transducer arrays, plane wave compounding, adaptive beamforming, minimum variance, generalized coherence factor

## Abstract

Plane wave compounding (PWC) is an effective modality for ultrafast ultrasound imaging. It can provide higher resolution and better noise reduction than plane wave imaging (PWI). In this paper, a novel beamformer integrating the two-dimensional (2-D) minimum variance (MV) with the generalized coherence factor (GCF) is proposed to maintain the high resolution and contrast along with a high frame rate for PWC. To specify, MV beamforming is adopted in both the transmitting aperture and the receiving one. The subarray technique is therefore upgraded into the sub-matrix division. Then, the output of each submatrix is used to adaptively compute the GCF using a 2-D fast Fourier transform (FFT). After the 2-D MV beamforming and the 2-D GCF weighting, the final output can be obtained. Results of simulations, phantom experiments, and in vivo studies confirm the advantages of the proposed method. Compared with the delay-and-sum (DAS) beamformer, the full width at half maximum (FWHM) is 90% smaller and the contrast ratio (CR) improvement is 154% in simulations. The over-suppression of desired signals, which is a typical drawback of the coherence factor (CF), can be effectively avoided. The robustness against sound velocity errors is also enhanced.

## 1. Introduction

Owing to the high efficiency, the low cost, and the non-invasive characteristics, ultrasound imaging has been an adequate technique for medical diagnosis [1,2,3]. However, the commonly adopted line scan mode for ultrasound imaging is limited to a low frame rate since it requires a series of focused beams for a single emission, which makes it difficult for the visualization of rapid tissue motions [4]. To realize ultrafast ultrasound imaging, plane wave imaging (PWI) is proposed, which utilizes a single pulse generation rather than a series of focused beams [5]. Due to the lack of focusing on pulse emissions, the frame rate is significantly increased while the imaging quality suffers a great degradation [5,6,7]. In addition, the unfocused beams cannot offer sufficient excitation energy for the region of interest (ROI). As a result, the signal-to-noise ratio (SNR) for the backscattered echo is lower than conventional methods.

To handle the shortcomings of PWI, plane wave compounding (PWC) is proposed to achieve a compromise between the high frame rate and the satisfactory imaging quality. The original concept can date back to 1981 [8]. Then, Lu and Cheng first introduced the spatial compounding of several steered plane waves [9,10,11]. By applying appropriate time delays for each transducer element, a plane wave with a particular steering angle is transmitted. After all firing events, a series of echoes can be recorded into a two-dimensional (2-D) data matrix, which is different from the 1-D echo vector in the PWI. Then, the echoes are compounded for the beamformed output. Until now, there are two categories of compounding methods. Incoherent PWC implements the incoherent summation of array signals to suppress the sidelobe effects [12,13]. Coherent PWC was originally proposed by Montaldo et al. [14], in which the received echoes from different emissions are compounded to enhance the imaging quality. However, the contradiction between the frame rate and the imaging quality is still an unsolved problem. More steered plane waves can bring better imaging performance while the frame rate will drop accordingly. Therefore, it is desirable to maintain the high quality ultrasound image with few plane wave transmissions.

Adaptive beamformers is an effective technique to improve the imaging quality. The most representative ones are the minimum variance (MV) based beamformers and the coherence factor (CF) based beamformers. The concept of MV was originally proposed in 1969 [15], and then introduced into ultrasound imaging [16,17]. The eigenspace-based minimum variance (ESBMV) beamformer is an optimization [18,19]. Recently, MV was also modified for the 2-D echo dataset in PWC, for instance, the multi-wave approach and the joint transmitting-receiving (JTR) methods [20,21]. MV contributes to a higher SNR and improves the resolution while the robustness could be decreased when compared with nonadaptive methods. The CF aims to attenuate the output signal and noise simultaneously to obtain a more accurate output [22]. There are different forms of CF beamformers in which the generalized coherence factor (GCF) is a typical application [23]. The advantages of the CF mainly occur in the higher contrast and less sidelobe effect while the major drawback of the CF is the over-suppression of desired signals. Until now, the CF has not been properly adapted for PWC. Previous works usually compute one GCF for each emission of PWC, which results in a high computational amount [24]. In consideration of these factors, maintaining both advantages of the MV and CF could be an attractive issue for enhancing ultrasound imaging quality.

In this paper, we integrate the GCF with a 2-D MV beamformer to obtain a high resolution and contrast at the same time. Specifically, the JTR adaptive weighting is first conducted using the PWC dataset. In this process, the spatial smoothing technique is adopted in not only the receiving aperture, but also the transmitting one, which means that both the spatial smoothing and the frame smoothing are used for the 2-D echo data. The subarray division is therefore upgraded into the sub-matrix division. After the MV weighting process, the output matrix can be obtained in which each element represents the adaptively weighted result of a single sub-matrix. The second step is to compound these sub-matrix results for the final output. We extend the definition of the GCF for the PWC, which means to replace the original fast Fourier transform (FFT) with a 2-D FFT. By modifying both the JTR method and the GCF beamformer specifically for the PWC, a considerably better imaging performance could be achieved.

The innovation point of our method comes from three aspects. First, the 2-D MV beamforming process is specially designed for the PWC. It is computationally inexpensive and helps improve the resolution. Second, the GCF, which originally works for a 1-D line scan mode or the PWI, is modified for the PWC modality. The proposed method calculates one GCF for each compounding image, not for each emission. Thus, the computational load is decreased. Besides, adopting the coherence weighting process could effectively reduce the noise level and enhance the imaging contrast. Last, but not least, the spatial smoothing technique plays an important role in the organic integration of the MV and CF. However, the combination of the MV and CF is not a brand-new conception [24,25,26,27]. There is some research related to the MV-CF combined methods in single transmission modalities, but few focus on the application in PWC [25,26,27]. The proposed method is conceptually different in the following ways. The 2-D echo data array is divided into several overlapped sub-matrices, as an upgraded version of the subarray technique. The MV process is averaged over these sub-matrices to enhance the robustness, while the GCF is calculated using the output of each sub-matrix rather than the recorded signal of each array element. As a result, the correlation and coherence information in the 2-D dataset is thoroughly utilized.

The remainder of this paper is divided into several sections. Section 2 introduces the background of existing techniques. Section 3 illustrates the algorithm of the proposed method in detail. Section 4 presents the simulated and experimental setup, along with the results of the proposed method and conventional methods. Section 5 provides the discussion and the final conclusion is given in Section 6.

## 2. Backgrounds

### 2.1. Mathematic Model of the Plane Wave Compounding

The PWC recombines a series of plane waves with different steering angles. The received echo signals can be recorded into a 2-D data matrix. One dimension stands for different array elements, which is called the receiving aperture. Another stands for different emissions, which can be regarded as the transmitting aperture [28]. If an *M*-element transducer array is used for ultrasound transmitting and receiving while *N* steered plane waves are fired in total, an *N* × *M* size echo data matrix could be obtained. After introducing an appropriate time delay into each channel, the final matrix can be expressed as follows:

(1) $$X\left(n\right)=\left[\begin{array}{cccc} x_{1,1}\left(n\right) & x_{1,2}\left(n\right) & \cdots & x_{1,M}\left(n\right)\\ x_{2,1}\left(n\right) & x_{2,2}\left(n\right) & \ldots & x_{2,M}\left(n\right)\\ \vdots & \vdots & \ddots & \vdots \\ x_{N,1}\left(n\right) & x_{N,2}\left(n\right) & \cdots & x_{N,M}\left(n\right) \end{array}\right],$$

where $x_{i,j}\left(n\right)$ is the echo signals recorded by the *j*th element when the *i*th steered plane wave is used for transmitting. *n* is the time step. In the conventional delay-and-sum (DAS) algorithm [29,30], all channel data is averaged through rows and columns for each imaging point:

(2) $$z_{DAS}\left(n\right)=\frac{1}{MN}\sum_{i=1}^{N}\omega_{T}\left(i\right)\sum_{j=1}^{M}\omega_{R}\left(j\right)x_{i,j}\left(n\right),$$

where $\omega_{R}$ is the receiving apodization window and $\omega_{T}$ is the transmitting one.

### 2.2. Minimum Variance Beamformers

The MV beamformer aims to minimize the output energy subject to the desired signal being undistorted [17]. For each single emission, the MV equation could be written as follows:

(3) $$z\left(n\right)=w^{H}\left(n\right)x\left(n\right),$$

where w(n) is the MV weighting vector and ${\left(\cdot \right)}^{H}$ denotes the conjugate transpose. While $x\left(n\right)={\left[x_{1}\left(n\right),x_{2}\left(n\right),\ldots ,x_{M}\left(n\right)\right]}^{T}$(${\left(\cdot \right)}^{T}$ is the matrix transpose) represents the signals recorded by different array elements with appropriate time delays. The MV process can be expressed as an optimization problem:

(4) $$\underset{w}{\min}\;w^{H}R\left(n\right)w,\;\mathrm{subject}\;\mathrm{to}\;w^{H}d=1,$$

since the x(n) is appropriately delayed, the steering vector, d, is equal to an all one vector. Here, R(n) is the covariance matrix.

The solution to (4) is:

(5) $$w_{MV}=\frac{R^{-1}d}{d^{H}R^{-1}d}.$$

The covariance matrix, R(n), is usually unknown and must be estimated:

(6) $$R\left(n\right)=E\left[x\left(n\right)x^{H}\left(n\right)\right].$$

To enhance the accuracy of the covariance matrix estimation, the spatial smoothing technique is proposed [31]. The transducer array is divided into several overlapped subarrays. Then, the covariance matrix of each subarray is averaged to decorrelate the highly correlated array signals:

(7) $$\bar{R}\left(n\right)=\frac{1}{M-L+1}\sum_{l=1}^{M-L+1}x_{l}\left(n\right)x_{l}^{H}\left(n\right),$$

where $x_{l}={\left[x_{l}\left(n\right),x_{l+1}\left(n\right),\ldots ,x_{l+L-1}\left(n\right)\right]}^{T}$ represents the *l*th subarray with a length of *L*. According to Refs. [31,32,33], the subarray length, *L*, should be smaller than M/2, a half of the array size. In addition, to avoid a singular covariance matrix, diagonal loading is also necessary. It is suggested to introduce a constant into the matrix: ${\bar{R}}_{DL}\left(n\right)=\bar{R}\left(n\right)+\varepsilon \cdot I$, where I is the identity matrix and the constant, ε, is often set to be Δ (smaller than 0.1) times of $\mathrm{trace},\;\left(\bar{R}\right)$ [16,17].

The modified weighting vector, $w_{MV}$, can be calculated by Equation (5) using the ${\bar{R}}_{DL}\left(n\right)$. Then, the beamformed output should also be averaged over subarrays, which means that Equation (3) should be rewritten into:

(8) $$z\left(n\right)=\frac{1}{M-L+1}\sum_{l=1}^{M-L+1}w_{MV}^{H}\left(n\right)x_{l}\left(n\right).$$

To further reduce the noise and increase the SNR, the ESBMV beamformer is proposed [18]. The covariance matrix, $\bar{R}$, is eigen-decomposed to project the $w_{MV}$ onto the signal subspace. This process can be illustrated as follows:

(9) $$\bar{R}=V\Lambda V^{H}=\sum_{k=1}^{K}\lambda_{k}v_{k}v_{k}^{H},$$

where $\Lambda =\mathrm{diag}\left[\lambda_{1},\lambda_{2},\ldots ,\lambda_{K}\right]$ represents all eigenvalues ($\lambda_{1}\geq \lambda_{2}\geq \ldots \geq \lambda_{K}$) and $v_{k}$ is the eigenvector corresponding to $\lambda_{k}$. Eigenvectors corresponding to first largest eigenvalues, which are larger than $\lambda_{thre}$, are selected to build the signal subspace:

(10) $$V_{s}=\left[v_{1},\ldots ,v_{thre}\right].$$

The ESBMV weight is calculated as:

(11) $$w_{ESBMV}=V_{s}{V_{s}}^{H}w_{MV}.$$

Then, the ESBMV output can be acquired using Equation (8).

### 2.3. Coherence Factor Beamformers

The CF is defined as the ratio between the coherent sum and the incoherent sum:

(12) $$\mathrm{CF}=\frac{{\left[\frac{1}{N}\sum_{i=1}^{N}x_{i}\left(n\right)\right]}^{2}}{\frac{1}{N}\sum_{i=1}^{N}\left[x_{i}^{2}\left(n\right)\right]}.$$

The GCF is a more efficient approach for the spatial spectrum based beamforming [23]. First, the *M*-point discrete Fourier transform (DFT) is carried out to obtain the Fourier spectrum. Usually, this process is implemented using the fast Fourier transform (FFT):

(13) p(k)=FFT(x(n)),

where *k* = 0 to *M* − 1 is the spatial frequency index. Then, the GCF can be defined as the ratio of the low-frequency energy to the total energy:

$$\mathrm{GCF}=\frac{energy\;within\;a\;low-frequency\;region}{total\;energy}$$

(14) $$=\frac{\sum_{k\in low-frequency\;region}{\left|p\left(k\right)\right|}^{2}}{\sum_{k=0}^{M-1}{\left|p\left(k\right)\right|}^{2}}.$$

Usually, the low-frequency region is selected by a cutoff frequency, $M_{0}$ (from $-M_{0}$ to $M_{0}$). For point targets, the $M_{0}$ is suggested to be 0, which means that only the direct current component is used for the GCF. For complicated situations, $M_{0}$ should be set to 1–3 to avoid artifacts [23].

## 3. Methods

### 3.1. Joint Transmitting-Receiving Beamforming

The JTR beamformer is a modified version of the MV beamformer for the PWC [21]. Conventional MV methods need to calculate one weighting vector for each firing event, thus the computational load would be extremely high. The JTR only needs two weights in total, the transmitting one and the receiving one, which brings a lower computational complexity (CC).

Taking account of the echo data matrix in Equation (1), the receiving covariance matrix is calculated first. Define $x_{Rx_{i}}\left(n\right)={\left[x_{i,1}\left(n\right),x_{i,2}\left(n\right),\ldots ,x_{i,M}\left(n\right)\right]}^{T}$ as the rows of the original matrix, which represents each single transmission. The receiving covariance matrix is estimated by averaging through not only the receiving subarrays, but also all transmitting events, which means:

(15) $${\bar{R}}_{Rx}\left(n\right)=\frac{1}{N\left(M-L_{1}+1\right)}\sum_{i=1}^{N}\sum_{l=1}^{M-L_{1}+1}x_{Rx_{i,l}}x_{Rx_{i,l}}^{H},$$

where $x_{Rx_{i,l}}={\left[x_{i,l}\left(n\right),x_{i,l+1}\left(n\right),\ldots ,x_{i,l+L_{1}+1}\left(n\right)\right]}^{T}$ is the *l*th subarray of the *i*th transmission with a length of $L_{1}$. The MV based weighting vector for the receiving aperture, $w_{Rx}={\left[w_{Rx}^{1},w_{Rx}^{2},\ldots ,w_{Rx}^{L_{1}}\right]}^{T}$, can be calculated using (5) or (11).

In consideration of the ultrasound reciprocity, the transmitting aperture weighting vector can be calculated using the same procedure. Define $x_{Tx_{j}}\left(n\right)={\left[x_{1,j}\left(n\right),x_{2,j}\left(n\right),\ldots ,x_{N,j}\left(n\right)\right]}^{T}$ as the columns of the original matrix, which represents each receiving element. The transmitting covariance matrix is estimated by averaging through the transmitting subarrays and all receiving elements, as well:

(16) $${\bar{R}}_{Tx}\left(n\right)=\frac{1}{M\left(N-L_{2}+1\right)}\sum_{j=1}^{M}\sum_{l=1}^{N-L_{2}+1}x_{Tx_{j,l}}x_{Tx_{j,l}}^{H},$$

where $x_{Tx_{j,l}}={\left[x_{l,j}\left(n\right),x_{l+1,j}\left(n\right),\ldots ,x_{l+L_{2}+1,j}\left(n\right)\right]}^{T}$ is the *l*th subarray of the array data recorded by the *j*th element with a length of $L_{2}$. The weighting vector for the transmitting aperture, $w_{Tx}={\left[w_{Tx}^{1},w_{Tx}^{2},\ldots ,w_{Tx}^{L_{2}}\right]}^{T}$, can also be obtained.

To accord with the spatial smoothing technique in both the transmitting and receiving dimension, the subarray division is upgraded into the submatrix division. The submatrix with a size of $L_{2}\times L_{1}$ can be described as follows:

(17) $${\hat{X}}_{i.j}=\left[\begin{array}{cccc} x_{i,j} & x_{i,j+1} & \cdots & x_{i,j+L_{1}-1}\\ x_{i+1,j} & x_{i+1,j+1} & \ldots & x_{i+1,j+L_{1}-1}\\ \vdots & \vdots & \ddots & \vdots \\ x_{i+L_{2}-1,j} & x_{i+L_{2}-1,j+1} & \cdots & x_{i+L_{2}-1j+L_{1}-1} \end{array}\right],$$

where $i\in \left[1,2,\ldots ,N-L_{2}+1\right],\;j\in \left[1,2,\ldots ,M-L_{1}+1\right]$ are the indexes of the submatrix. The JTR beamformed output of a single submatrix can be calculated by:

(18) $$z_{i,j}\left(n\right)=w_{Tx}^{T}\hat{X}\left(n\right)w_{Rx}.$$

In original JTR implementation, the final output is obtained by the averaged superposition of all $z_{i,j}\left(n\right)$ [21], which still leaves room for improvements.

### 3.2. 2-D generalized Coherence Factor Weighting

Taking account of the JTR output matrix:

(19) $$Z\left(n\right)=\left[\begin{array}{cccc} z_{1,1} & z_{1,2} & \cdots & z_{1,M-L_{1}+1}\\ z_{2,1} & z_{2,2} & \ldots & z_{2,M-L_{1}+1}\\ \vdots & \vdots & \ddots & \vdots \\ z_{N-L_{2}+1,1} & z_{N-L_{2}+1,2} & \cdots & z_{N-L_{2}+1,M-L_{1}+1} \end{array}\right].$$

Here, we intend to use the GCF to constrain the final output. A simple solution is to arrange all elements in Equation (19) in a row and then implement the FFT. However, since the echo data in Equation (19) is from different firing events, the 1-D FFT cannot obtain the accurate low-frequency component. Another alternative is to calculate one GCF for each firing event, which means one GCF for each row in Equation (1) [24]. This process is more accurate, but could bring a high computational load.

In this paper, we propose to use the 2-D FFT of the output matrix (19). We can acquire the 2-D spatial spectrum of the original signal:

(20) $$P\left(f\right)=\mathrm{FFT}\left[Z\left(n\right)\right]=\left[\begin{array}{cccc} p_{1,1} & p_{1,2} & \cdots & p_{1,M-L_{1}+1}\\ p_{2,1} & p_{2,2} & \ldots & p_{2,M-L_{1}+1}\\ \vdots & \vdots & \ddots & \vdots \\ p_{N-L_{2}+1,1} & p_{N-L_{2}+1,2} & \cdots & p_{N-L_{2}+1,M-L_{1}+1} \end{array}\right],$$

where $p_{i,j}$ stands for the corresponding frequency domain component. Similar to the definition of the 1-D GCF, the 2-D GCF is also calculated as the ratio between the low-frequency energy to the total energy, but this time the low-frequency component is selected in both dimensions, which means:

$$GCF_{2}=\frac{\sum_{k\in low-frequency\;region}{\left|p_{k_{1},k_{2}}\right|}^{2}}{\sum {\left|P\right|}^{2}}$$

(21) $$=\frac{\sum_{k_{1}=0}^{M_{1}}\sum_{k_{2}=0}^{M_{2}}{\left|p_{k_{1},k_{2}}\right|}^{2}}{\sum {\left|P\right|}^{2}},$$

where $M_{1}$ and $M_{2}$ are the cut-off frequency in the transmitting dimension and the receiving dimension to define the 2-D low-frequency region. Using the 2-D GCF, the final output of the proposed method could be obtained:

(22) $$z_{out}=GCF_{2}\times sum\left(Z\left(n\right)\right).$$

Additional remarks should be specified here that there are two distinct differences between the modified GCF and the conventional one. First, the ${\mathrm{GCF}}_{2}$. adopts the 2-D FFT, and the 2-D low-frequency components are selected, which accords with the 2-D echo data matrix of the PWC. Second, the JTR beamformed output of all submatrices (19), instead of the original echo data matrix (1), is used as the time-domain signal of the FFT. Since different rows in Equation (1) come from different emissions, adopting a 2-D FFT on (1) could bring spatial frequency errors. Therefore, the spatial smoothing technique is adopted to divide the original matrix (1) into several overlapped sub-matrices and then these sub-matrices are MV beamformed. As a result, the new FFT on Equation (19) could bring more accuracy in the frequency domain than the conventional one, which may result in a better imaging performance.

### 3.3. Implementation Summary

A brief implementation summary of the proposed algorithm is given here to illustrate the method in a clear way:

(1) Calculate time delays for each channel of different plane wave firing events. The echo data matrix (1) can be obtained after the calculation.
(2) Compute the JTR weighting vector, $w_{Tx}$ and $w_{Rx}$, using Equations (5), (15), and (16). The JTR beamformed output matrix (19) can be acquired from Equation (18).
(3) Use the 2-D FFT to get the spatial spectrum and then calculate the 2-D GCF using Equation (21). The final output of the proposed algorithm is obtained from (22).
(4) Repeat the procedure I to III over each imaging pixel to generate the final B-mode image.

## 4. Experiments and Results

### 4.1. Experimental Setup

**Figure 1:**
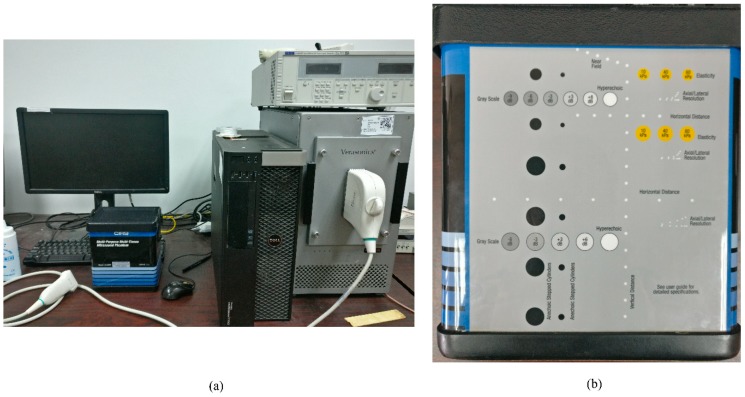
(**a**) The Verasonics Vantage Advantage system in our laboratory (V1, Verasonics, Redmond, WA, USA). (**b**) The commercially general-purpose multi-tissue phantom (Model 040GSE, CIRS, Norfolk, VA, USA).

The proposed method was evaluated through simulations, phantom experiments, and in vivo studies. The Matlab simulation tool, Field II, was used to acquire simulated data [34,35]. The Verasonics ultrasound platform (V1, Verasonics, Redmond, WA, USA), as shown in Figure 1a, was used to acquire the phantom and in vivo data. For both simulations and experiments, a 5-MHz, 128-element transducer array with a 0.3 mm pitch was used (L11-4v, Verasonics, Redmond, WA, USA). The excitation pulse was a two-cycle sinusoid and the sampling rate was 40 MHz. For the PWC implementation, 49 steered plane waves at a 0.5° interval ranging from −12° to 12° were emitted in total.

Three point targets at the depths of 30 mm, 35 mm, and 40 mm were used for the point spread function (PSF) simulation. Two 2.5-mm radius circular anechoic cysts at the depths of 37 mm and 52 mm were adopted for the cyst simulation. For phantom experiments, a CIRS calibration phantom (Model 040GSE, CIRS, Norfolk, VA, USA), as shown Figure 1b, was used to acquire both hyperechoic point data and anechoic cyst data. Further information about this phantom can be found through the CIRS official homepage or this website: http://www.cirsinc.com/products/modality/67/multi-purpose-multi-tissue-ultrasound-phantom/. A 28-year-old male volunteer helped us to obtain the in vivo data of two human carotid arteries, a human thyroid and a human parotid.

For B-mode image evaluations, the simulated and experimental results using the proposed JTR-MV GCF and JTR-ESBMV GCF are presented along with results of the DAS, the CF, the JTR MV, and the JTR ESBMV. First, the DAS result is shown as the reference of other methods. Then, the CF result shows the effect of only using CF-based methods while the JTR results show the influence of only using MV-based methods. At last, the results of the proposed method are given to validate the effectiveness of the integration between JTR and GCF. An additional experiment is also implemented to investigate the influence of sound velocity errors on different beamformers. A single point is simulated with an overestimation of the sound velocity by 0%, 2%, 5%, and 10%. Results of the DAS, the CF, the JTR-MV, the JTR-ESBMV, the JTR-MV GCF, and the JTR-ESBMV GCF are shown together.

### 4.2. Parameters and Evaluation Metrics

The parameters used in simulations and experiments are carefully selected. The subarray length used for the spatial smoothing is set to 0.3 times the aperture size to make a compromise between the imaging quality and the calculation time [36]. To specify, $L_{1}=0.3M=0.3\times 128\approx 38$ and $L_{2}=0.3N=0.3\times 49\approx 15$. The ESBMV threshold, β, is set to 0.02 to define the signal subspace in which eigenvalues are larger than β times the largest eigenvalue [21,36]. The cut-off frequencies of the GCF are $M_{1}=M_{2}=0$ for point targets and $M_{1}=M_{2}=1$ for cysts and in vivo tissues according to Ref. [23]. A large cut-off frequency could avoid dark artifacts in the speckle region. The f-number is 1.4 with a rectangular window and the diagonal loading factor Δ is set to 0.01.

For quantitative assessment, the evaluation metrics used in the following section are the full width at half maximum (FWHM) (the −6 dB bandwidth of the mainlobe) for point targets and the contrast ratio (CR), and the contrast-to-noise ratio (CNR) for cyst targets. The CR and CNR can be calculated by:

(23) $$\mathrm{CR}=20\log_{10}(\mu_{b}/\mu_{c}),$$

(24) $$\mathrm{CNR}=\frac{\left|\mu_{b}-\mu_{c}\right|}{\sqrt{\sigma_{b}^{2}+\sigma_{c}^{2}}},$$

where $\mu_{b}$ and $\mu_{c}$ are the mean magnitudes of the signal inside the speckle and cyst, and $\sigma_{b}$ and $\sigma_{c}$ are the standard deviations of the magnitude in the speckle and cyst, respectively.

All transducer and acquisition parameters along with processing parameters are given in Table 1.

### 4.3. Simulated Study

**Figure 2:**
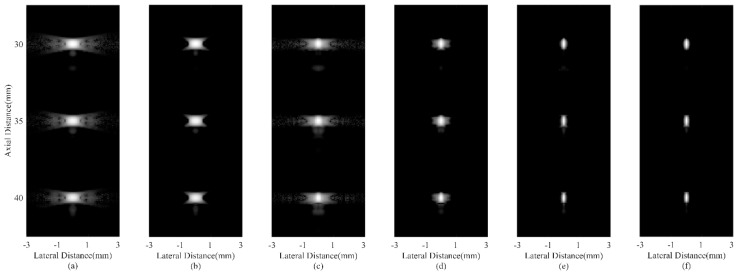
Simulated point targets using different beamformers: (**a**) DAS, (**b**) CF, (**c**) JTR-MV, (**d**) JTR-MV GCF, (**e**) JTR-ESBMV, (**f**) JTR-ESBMV GCF. All images are shown with a dynamic range of 60 dB.

Simulated point target images are shown in Figure 2. Figure 2a is the DAS result with visually obvious sidelobes. In Figure 2b, the CF shows its effectiveness in suppressing sidelobe noises. Figure 2c,e are results of the MV based adaptive beamformers, which indicate that the MV can enhance the imaging resolution while the ESBMV could bring better noise reduction than the conventional MV. The JTR-MV GCF and JTR-ESBMV GCF results are presented in Figure 2d,f respectively. From the comparison between Figure 2c,e and Figure 2d,f, it can be seen that the proposed method achieves a narrower mainlobe width than the JTR method. The narrow mainlobe and low sidelobes indicate that the proposed method could bring a high resolution.

**Figure 3:**
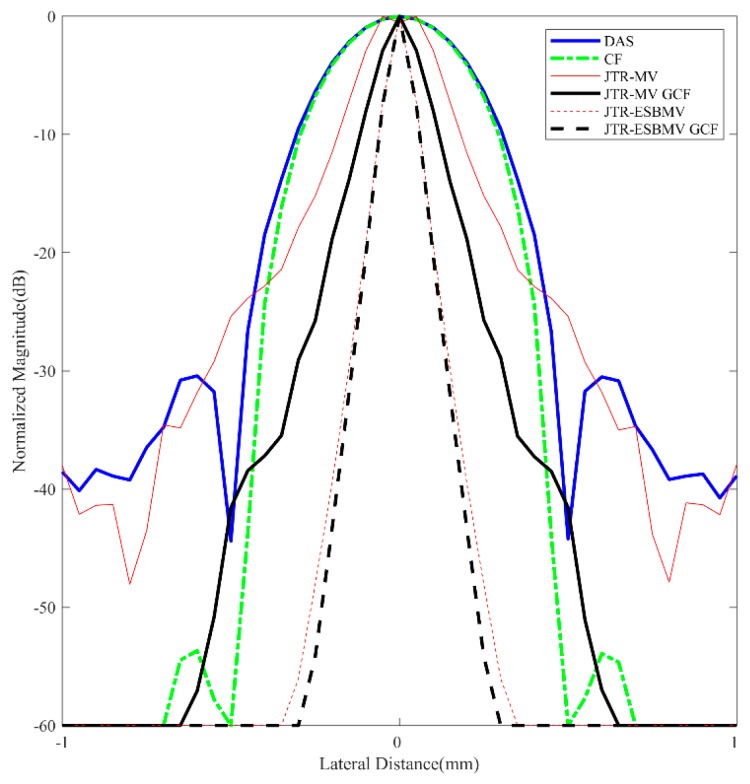
Lateral variations at *z* = 30 mm in simulated point images.

For further comparisons, lateral variation plots at *z* = 30 mm of different beamformers are shown in Figure 3 while the corresponding FWHMs are given in Table 2. The proposed JTR-ESBMV GCF achieves the best performance with a 90% smaller FWHM than the conventional DAS beamformer, which validates the resolution improvement by the proposed method.

**Figure 4:**
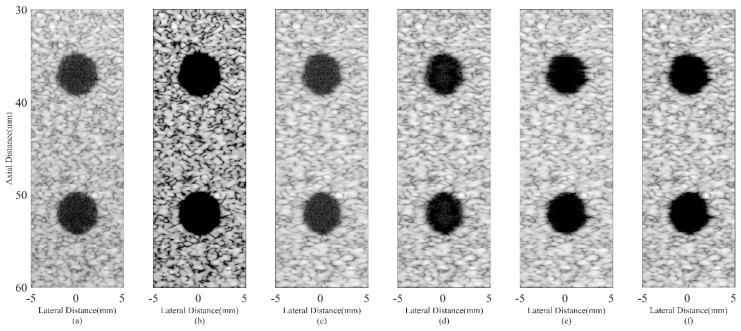
Simulated anechoic cysts using different beamformers: (**a**) DAS, (**b**) CF, (**c**) JTR-MV, (**d**) JTR-MV GCF, (**e**) JTR-ESBMV, (**f**) JTR-ESBMV GCF. All images are shown with a dynamic range of 60 dB.

Simulated anechoic cyst images are shown in Figure 4. In Figure 4a, noises are still at a high level inside cysts, which results from the sidelobes of background speckles. The CF result in Figure 4b obtains a satisfactory noise reduction inside cysts. However, signals inside the speckle region are also over-suppressed and the speckle patterns are severely damaged. It is due to the distortion characteristic of the CF method. The JTR method can obtain a better speckle performance and help remove the noise slightly as shown in Figure 4c,e. In comparison, the proposed method can significantly remove the noise inside cysts, which brings a more distinct cyst boundary. While the speckle patterns are still well preserved with less dark spots thanks to the modified GCF. The results confirm that the proposed method has the potential of maintaining the high contrast and good speckle performance at the same time.

Numerical results of the CR and CNR for both cysts are shown in Table 3. The speckle region at the same depth with the cyst is selected for the CR and CNR calculation in order to avoid signal attenuations. From the results, it is shown that the CF can bring an extremely high CR since it can effectively suppress the noise while the CNR suffers a great degradation due to the damaged speckle. On the contrary, MV based methods could bring a higher CNR, but the CR improvement is comparably little. In comparison, the proposed method can significantly increase the CR while the CNR drops a little when compared with the JTR method, but is still at a similar level with the DAS. Sidelobe noises inside the cyst can be effectively removed by the proposed method, which results in a 154% higher CR than the DAS method.

### 4.4. Experimental Phantom Study

**Figure 5:**
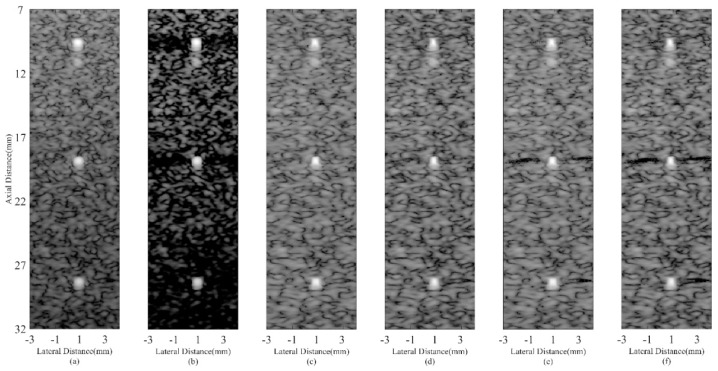
Experimental point targets phantom using different beamformers: (**a**) DAS, (**b**) CF, (**c**) JTR-MV, (**d**) JTR-MV GCF, (**e**) JTR-ESBMV, (**f**) JTR-ESBMV GCF. All images are shown with a dynamic range of 60 dB.

The wire target phantom results are shown in Figure 5, which are similar to the simulated results presented before. Both the MV methods and the CF ones can narrow the mainlobe compared with the DAS beamformer. However, the speckle performance of the CF is unsatisfactory. The proposed method can obtain a narrow mainlobe and a smooth speckle simultaneously. The deep region performance is also better than the DAS method.

**Figure 6:**
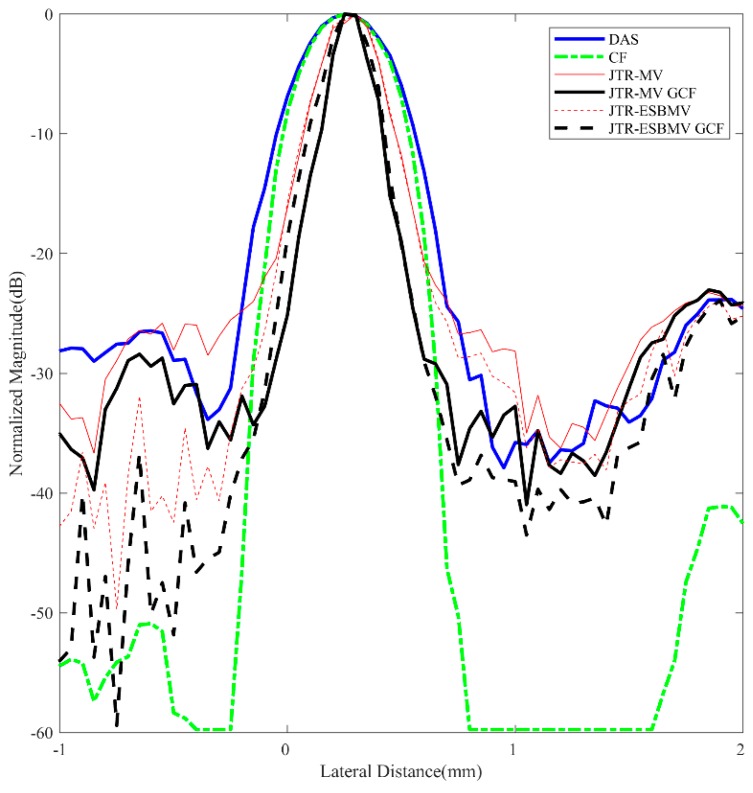
Lateral variations at *z* = 9 mm in experimental point images.

Figure 6 gives the lateral variation across the depth of 9 mm. Table 4 provides the statistical results of the FWHM. The proposed JTR-ESBMV GCF obtains a 51% smaller FWHM than the DAS beamformer.

**Figure 7:**
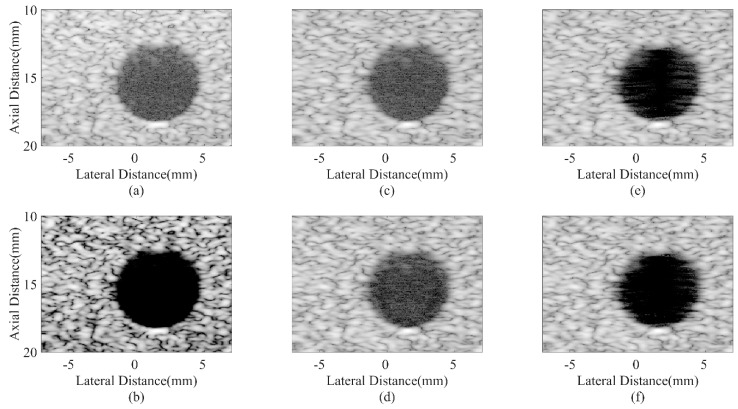
Experimental anechoic cyst phantom using different beamformers: (**a**) DAS, (**b**) CF, (**c**) JTR-MV, (**d**) JTR-MV GCF, (**e**) JTR-ESBMV, (**f**) JTR-ESBMV GCF. All images are shown with a dynamic range of 60 dB.

Results of the anechoic cyst phantom are presented in Figure 7. The advantages of the proposed method mainly occur in better noise reduction, clearer cyst edges, and better-preserved speckle patterns. The CRs and CNRs are given in Table 5 as the evaluation metrics. The experimental results are similar to the simulated ones, in which the JTR-ESBMV GCF achieve the highest CR (111% higher than the DAS one). In addition, the CNR is also a little better than that of the DAS beamformer, but slightly worse than the JTR methods. This indicates that the most prominent advantage of the proposed method is the high contrast.

### 4.5. In Vivo Study

**Figure 8:**
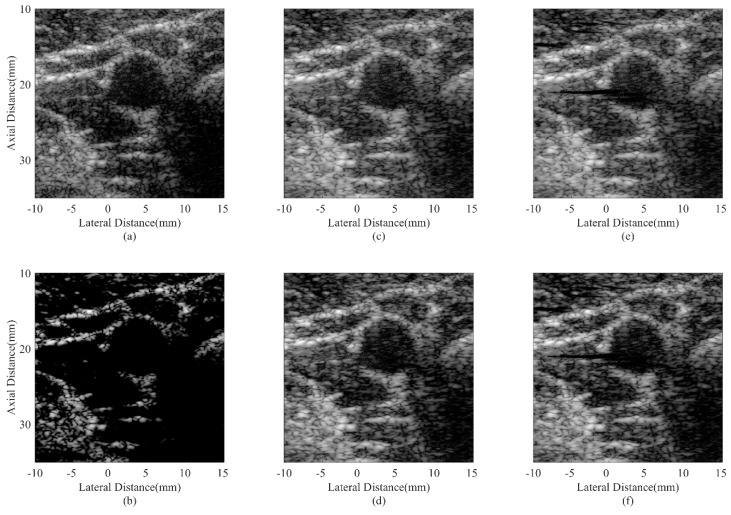
In vivo human carotid artery images using different beamformers: (**a**) DAS, (**b**) CF, (**c**) JTR-MV, (**d**) JTR-MV GCF, (**e**) JTR-ESBMV, (**f**) JTR-ESBMV GCF. All images are shown with a dynamic range of 60 dB.

**Figure 9:**
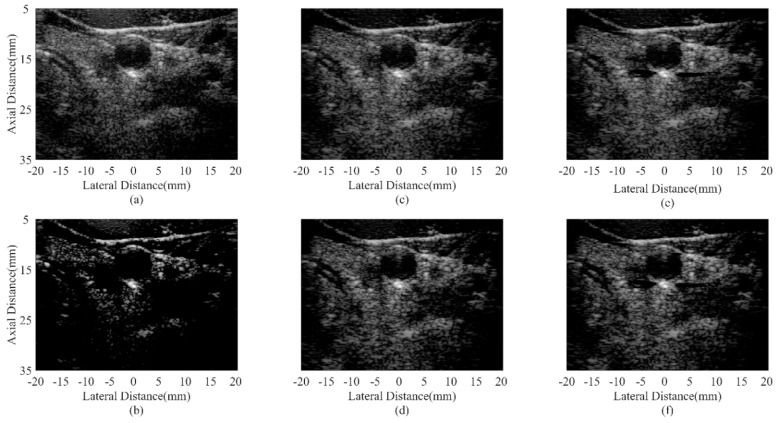
In vivo human carotid artery images using different beamformers: (**a**) DAS, (**b**) CF, (**c**) JTR-MV, (**d**) JTR-MV GCF, (**e**) JTR-ESBMV, (**f**) JTR-ESBMV GCF. All images are shown with a dynamic range of 60 dB.

Figure 8 and Figure 9 show in vivo results from two human carotid arteries. The anatomic structure of a blood vessel is similar to an anechoic cyst inside the speckle region. Therefore, the most interesting issues are noises inside the blood vessel and edges of the artery wall. From the results, it could be observed that noises inside the vessel are obvious with the DAS beamformer. Adaptive beamformers can help reduce the noise level while the MV can further preserve the outside-vessel region. The CF result suffers from a low intensity due to the over-suppression of the desired signal. Using the proposed method, the hyperechoic structures can be better distinguished and the noise reduction in anechoic regions is significantly enhanced. Over-suppression is also avoided.

For quantitative assessment, the CRs and CNRs are calculated using the anechoic region inside the blood vessel and the background tissues in Figure 8. Results of different beamformers are given in Table 6. The proposed JTR-ESBMV GCF achieves a high performance in the CR. Statistical results further validate the effectiveness of the proposed method in enhancing the imaging quality.

**Figure 10:**
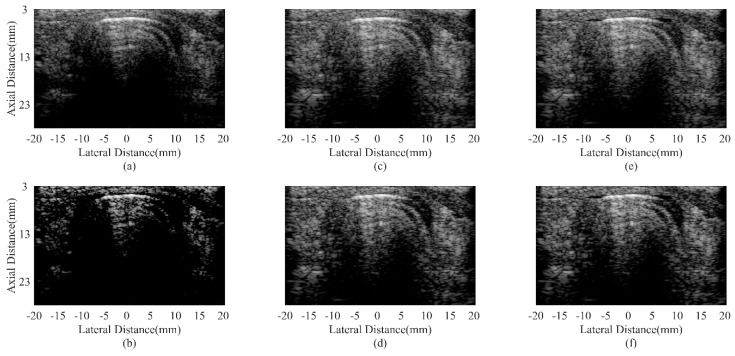
In vivo human thyroid images using different beamformers: (**a**) DAS, (**b**) CF, (**c**) JTR-MV, (**d**) JTR-MV GCF, (**e**) JTR-ESBMV, (**f**) JTR-ESBMV GCF. All images are shown with a dynamic range of 60 dB.

The human thyroid results are given in Figure 10. Compared with the DAS result in Figure 10a, both the JTR method and the proposed method can preserve the speckle patterns inside the thyroid, especially at the center of these images. However, the CF result suffers a low intensity and dark artifacts. It is due to the over-suppression of the desired signals caused by a small CF. In comparison with the JTR method, the contrast around the hyperechoic region is enhanced in Figure 10f. The over-suppression could also be effectively avoided. As a result, a better visualization of the thyroid structure can be obtained with the proposed method.

**Figure 11:**
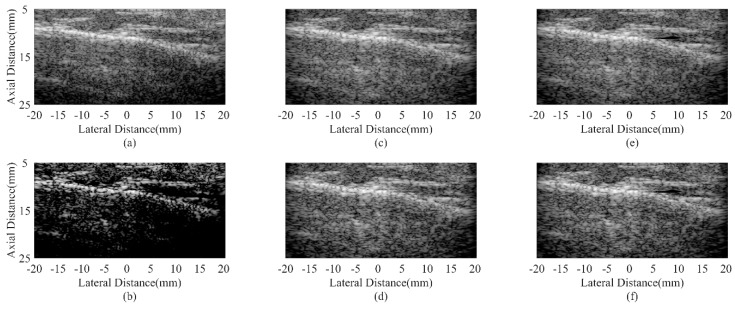
In vivo human parotid images using different beamformers: (**a**) DAS, (**b**) CF, (**c**) JTR-MV, (**d**) JTR-MV GCF, (**e**) JTR-ESBMV, (**f**) JTR-ESBMV GCF. All images are shown with a dynamic range of 60 dB.

Figure 11 presents in vivo results of a human parotid. Similar to the thyroid results, the hyperechoic structure is more distinguishable with the proposed JTR-ESBMV GCF beamformer. In Figure 11b, the whole image is hard to recognize because of the low intensity. The speckle patterns are severely damaged by the CF. In Figure 11e,f, speckles are well preserved, and the boundary between the hyperechoic region and the speckle is also better defined.

All in vivo results demonstrate that the proposed method is effective in enhancing the imaging details and preserving the imaging structure. A better visualization of anatomical structures can be obtained, which could contribute to future medical diagnosis.

### 4.6. Robustness to Sound Velocity Inhomogenetities

**Figure 12:**
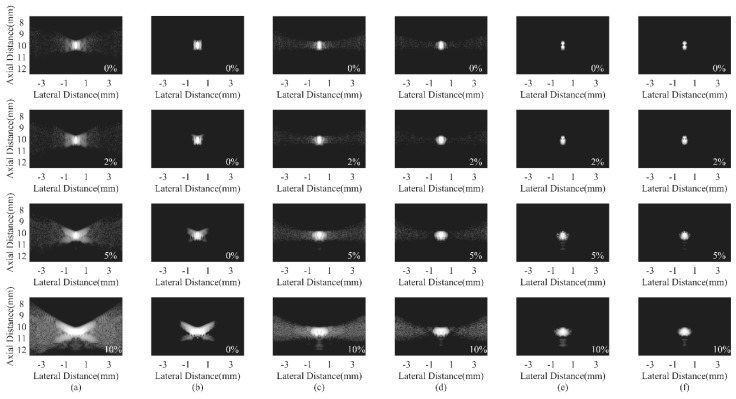
Simulated PSFs under different sound velocity errors using different beamformers: (**a**) DAS, (**b**) CF, (**c**) JTR-MV, (**d**) JTR-MV GCF, (**e**) JTR-ESBMV, (**f**) JTR-ESBMV GCF. The error rate is indicated in a white font in each image. All images are shown with a dynamic range of 60 dB.

An additional experiment is specially conducted to study the influence of sound velocity inhomogenetities on different beamformers. When ultrasound waves propagate through different kinds of tissues, such as skins and blood, the sound velocity difference of these tissues could bring calculation errors for the propagation time. In particular, for the PWC, sound velocity errors also result in an inaccurate steering vector. The high performance of conventional beamformers could be degraded as a result [37,38]. Figure 12 shows the simulated PSFs with an 0%, 2%, 5%, and 10% overestimation of the sound velocity using the DAS, the CF, the JTR-MV, the JTR-MV GCF, the JTR-ESBMV, and the JTR-ESBMV GCF. As shown in Figure 12a, the sound velocity errors cause a wide mainlobe and severe sidelobe artifacts. The CF method could effectively suppress the sidelobes, which validates the noise suppressing advantage of the CF. Using the JTR method, sidelobes are partly removed. With the proposed JTR-ESBMV GCF, the sound velocity errors show little influence on the PSF, which can be proved from the comparison between different overestimations in Figure 12f. To conclude, the proposed beamformer achieves a high robustness to sound velocity inhomogeneities.

## 5. Discussion

The proposed method is specially designed for the PWC. The MV beamformer is adopted on both apertures to improve the resolution. After obtaining the MV beamformed output matrix, the modified 2-D GCF is used to constrain the final output for the better noise reduction. From the simulations and experiments, the proposed method shows its potential in achieving the high resolution and contrast simultaneously. The innovations of this method can be summarized into three aspects. First, the MV beamforming process is conducted on both apertures. Compared with conventional MV implementations on the receiving aperture, our method fully utilizes the 2-D data information. As a result, the high resolution can be expected. Second, the GCF is specially modified for the 2-D echo data matrix. It is proposed to use the 2-D FFT for the GCF calculation. The low-frequency component is defined with more accuracy than the original GCF. Thus, the improved GCF could bring better noise reduction and avoid over-suppression of desired signals. Third, the subarray division in the spatial smoothing technique is upgraded into the sub-matrix division, which is applied into both the MV process and the GCF one. Specifically, using the spatial smoothing, the MV helps obtain the beamformed outputs of different sub-matrices. After that, the 2-D GCF is computed using the MV output rather than the original echo data matrix. Since the sub-matrices are overlapped, the robustness is therefore enhanced and a smooth speckle performance can be obtained. In consideration of these factors, the high performance of the proposed method is reasonably convincing.

The proposed method has a comparably lower CC than conventional adaptive beamformers. For each PWC with *N* emissions, it only needs to calculate two MV weights and one GCF while single frame methods require *N* MV weights and *N* GCFs [16,24]. Due to the application of the FFT, the GCF calculation has a much lesser computational amount than the MV process and can be neglected. Therefore, the computational load mainly comes from the dual aperture MV process. For receiving aperture beamforming, the computation for ESBMV weights has a complexity of $O\left(L_{1}^{3}\right)$ while for transmitting aperture, the number becomes $O\left(L_{2}^{3}\right)$ [19,21,36]. Specifically, for the $L_{1}\times L_{1}$ size sub-matrix in the receiving aperture, the matrix inversion and eigen-decomposition require $2/3L_{1}^{3}$ and $21L_{1}^{3}$ floating operations, respectively. Likewise, the same calculation can be done for the transmitting aperture. For the GCF calculation, in consideration of the FFT technology, the 2-D Fourier transform of the $\left(N-L_{2}+1\right)\times \left(M-L_{1}+1\right)$ size matrix requires $\left(N-L_{2}+1\right)\log_{2}\left(N-L_{2}+1\right)+\left(M-L_{1}+1\right)\log_{2}\left(M-L_{1}+1\right)$ operations. The total computational amount is $2/3L_{1}^{3}+21L_{1}^{3}+2/3L_{2}^{3}+21L_{2}^{3}+\left(N-L_{2}+1\right)\log_{2}\left(N-L_{2}+1\right)+\left(M-L_{1}+1\right)\log_{2}\left(M-L_{1}+1\right)$, only slightly higher than that of the JTR method. In addition, it can be inferred that there is a positive correlation between the calculation amount and the subarray size. It accords with the statement presented earlier that a larger subarray size could bring better noise reduction and lower sidelobes at a cost of more calculations. In consideration of these factors, the proposed method is promising for ultrafast ultrasound imaging.

Thanks to the wide application of the graphics processing unit (GPU), the real-time MV implementation could be realized [39,40]. However, the static imaging and dynamic videos are quite different. Specifically, in practical situations, tissue motions and environment disturbance could severely degrade the imaging quality. In addition, whether the proposed method introduces frame-to-frame artefacts is worth investigation. Therefore, it would be valuable to study the video data of a real-time implementation. A comparison between the dynamic and static imaging is also necessary. Future studies will focus on the real-time in vivo application of the proposed method. Related techniques in audio processing will also be taken into consideration [41].

## 6. Conclusions

In this paper, we proposed a novel beamformer for PWC, which integrates the 2-D MV method with the GCF. Both the MV and the GCF were modified for the PWC imaging mode to maintain the high resolution and contrast. Results of simulations, phantom experiments, and in vivo studies validate that the proposed method was effective in enhancing the imaging quality. A high contrast and a high robustness against the sound velocity error could be obtained. In conclusion, the proposed method could be promising for maintaining the satisfactory imaging quality for ultrafast medical ultrasound imaging. Future works will focus on the real-time application of the proposed method, especially in in vivo situations. The tissue motion compensation technique will be taken into consideration to improve the dynamic imaging quality.

## Figures and Tables

**Table 1:** Transducer and processing parameters.

Parameter	Value	Parameter	Value
Sound speed *c* Number of elements *M* Transducer center frequency $f_{0}$ Element pitch Cycles in emitted pulse Range of steering angles Number of plane waves *N*	1540 m/s 128 5 MHz 0.3 mm 2 −12°–12° 49	Sampling frequency $f_{s}$ Receive F-number Diagonal loading factor Δ Receiving subarray length $L_{1}$ Transmitting subarray length $L_{2}$ ESBMV threshold β Cut-off frequency of GCF $M_{1},M_{2}$	40 MHz 1.4 0.01 38 (0.3*M*) 15 (0.3*N*) 0.02 0 (point)/1 (others)

**Table 2:** FWHM for the simulated point target at *z* = 30 mm.

Beamformer	FWHM (mm)
DAS	0.40
CF JTR-MV JTR-MV GCF JTR-ESBMV JTR-ESBMV GCF	0.37 0.13 0.12 0.05 0.04

**Table 3:** CR and CNR for simulated anechoic cysts.

Beamformer	CR (dB)	CNR
DAS	30.61/33.98	1.58/1.67
CF JTR-MV JTR-MV GCF JTR-ESBMV JTR-ESBMV GCF	81.23/85.34 31.04/33.50 38.06/40.65 54.08/61.44 76.50/86.36	1.01/1.26 1.65/1.74 1.59/1.67 1.63/1.72 1.58/1.66

**Table 4:** FWHM for the experimental point target at *z* = 9 mm.

Beamformer	FWHM (mm)
DAS	0.51
CF JTR-MV JTR-MV GCF JTR-ESBMV JTR-ESBMV GCF	0.48 0.32 0.20 0.30 0.25

**Table 5:** CR and CNR for experimental anechoic cysts.

Beamformer	CR (dB)	CNR
DAS	30.32	1.76
CF JTR-MV JTR-MV GCF JTR-ESBMV JTR-ESBMV GCF	76.77 29.87 33.27 50.97 64.01	1.13 1.82 1.74 1.86 1.78

**Table 6:** CR and CNR for a human carotid artery.

Beamformer	CR (dB)	CNR
DAS	20.19	1.57
CF JTR-MV JTR-MV GCF JTR-ESBMV JTR-ESBMV GCF	59.69 24.50 27.11 25.23 28.03	0.89 1.60 1.55 1.61 1.56

## Abbreviations

The following abbreviations are used in this manuscript:

2-D	Two-dimensional
CC	Computational complexity
CF	Coherence factor
CNR	Contrast-to-noise ratio
CR	Contrast ratio
DAS	Delay-and-sum
DFT	Discrete Fourier transform
ESBMV	Eigenspace-based minimum variance
FFT	Fast Fourier transform
FWHM	Full width at half maximum
GCF	Generalized coherence factor
JTR	Joint-transmitting-receiving
MV	Minimum variance
PSF	Point spread function
PWC	Plane wave compounding
PWI	Plane wave imaging
ROI	Region of interest
SNR	Signal-to-noise ratio
